# Solitary, adult-onset, intraosseous myofibroma of the rib: a case report and literature review

**DOI:** 10.1177/2050313X231182791

**Published:** 2023-06-21

**Authors:** Anne Weidlich, Jessica Pablik, Klaus-Dieter Schaser, Doreen Winkler, Elisabeth Mehnert, Hagen Fritzsche

**Affiliations:** University Hospital Carl Gustav Carus Dresden, Dresden, Germany

**Keywords:** Myofibroma, intraosseous, adult-onset, solitary, rib, chest

## Abstract

Myofibromas are rare benign tumors with myofibroblastic origin. They occur especially in cutis and subcutaneous tissue of the head and the neck, less frequently on the extremities. Myofibromas grow very slowly and are often painless, which is why patients often present relatively late. In the literature, there were many reports about intraosseous myofibromas of the craniofascial bones but reports of the trunk and extremities in adults are very rare. The authors present a very rare case of an intraosseous myofibroma of the ribs resulting in pathological fracture, including a research of literature from other cases of intraosseous myofibromas of the trunk or extremities.

## Introduction

Myofibromas are rare benign tumors with myofibroblastic origin. They occur especially in cutis and subcutaneous tissue of the head and the neck, they are observed less frequently on the extremities.^1–3^ They occur most frequently in children and infants up to the age of 2 years and less frequently in adults.^1,2,4^ There are solitary or multicentric lesions and, in rare cases, also affect the visceral organs.^2^

In children, multicentric myofibromas are often associated with infantile myofibromatosis^1^ and do not differ histopathologically from solitary lesions. Infantile myofibromatosis is caused by a PDGFRB (platelet-derived growth factor receptor beta) gene mutation with manifestations ranging from solitary or multiple tumors of the skin to generalized type with tumors of the skin and visceral involvement. A precise family history and physical examination often lead to the diagnosis, with histopathology being the gold standard to confirm the diagnosis. An autosomal dominant and recessive pattern of inheritance has been described, but most cases occur sporadically.^5^ Visceral lesions mean a very unfavorable course with high mortality. If the internal organs are not involved, the prognosis is very favorable, with often spontaneous regression.

Since myofibromas typically grow very slowly and are often painless, patients often present relatively late.^2^ Patients usually present with painless soft tissue swelling or, in the case of intraosseous lesions, occasionally with pain or pathological fractures.^1,2,6^ Imaging of intraosseous myofibromas often shows a sharply demarcated, nonspecific lytic mass with a sclerotic border, with or without pathologic fracture.^1,2^ Radiographically, myofibromas particularly resemble non-ossifying fibromas, fibrous dysplasia, and histiocytosis. But enchondromas, hemangiomas, aneurysmal bone cysts, and an infectious process can also look similar.^1,2^

## Case report

### Anamnese

We report on a 65-year-old female patient who was referred to our center with an intraosseous mass of the eighth rib and history of a fall on the left chest wall 2 months ago. A diagnostic outpatient chest radiograph was performed for pain in the left hemithorax, revealing a pathological fracture with an expansive osteolysis and unclear intraosseous of the eighth rib on the left lateral side. Following additional computed tomography (CT) and magnetic resonance imaging (MRI) scans, the patient was referred to our sarcoma center.

### Clinical findings

At the time of presentation, almost 3 months after the fall, the patient no longer reported any symptoms. Pain or shortness of breath was denied, as well as B symptoms or a known tumor disease. The clinical examination showed normal soft tissues on the left hemithorax without triggerable pressure or chest compression pain, a tumor in the area of the ribs/chest wall was not palpable.

### Imaging

**Figure 1.**
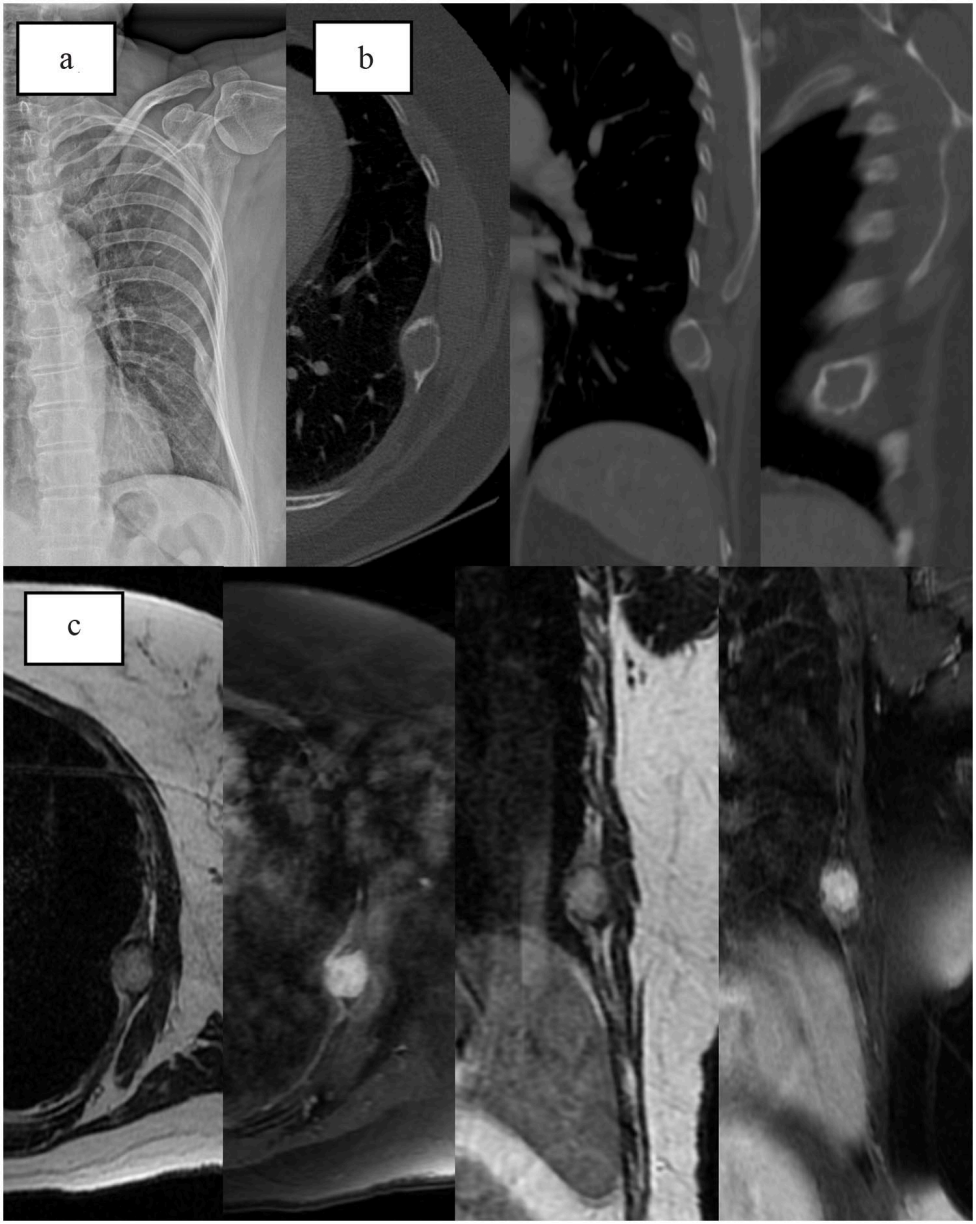
(a) X-ray of the left-sided chest wall in anterior-posterior plane. (b) CT scan of the chest wall in axial, coronal, and sagittal view. (c) MRI of the chest wall in axial (T2, T1 fs with gadolinium) and coronal (T2 and T1 fs with gadolinium) view.

External X-ray diagnostics showed a pathological fracture of the eighth left lateral rib with an unclear osteolysis (Figure 1(a)). The CT scan of the ribs showed an expansive, osteolytic intraosseous lesion, 3 × 2 cm in size, with a sclerotic rim and accompanying pathological fracture of the eighth rib on the left lateral side (Figure 1(b)). Similarly, the MRI of the thorax demonstrated evidence of a contrast medium–enhancing lesion without a significant surrounding reaction (Figure 1(c)). A staging CT of the thorax, abdomen, and pelvis showed an arterial hypervascularized lesion in the liver, which was constant in size in the CT follow-up controls. There was no evidence of further lesions. No abnormalities were found in the laboratory blood chemistry examination.

### Histological findings

**Figure 2.**
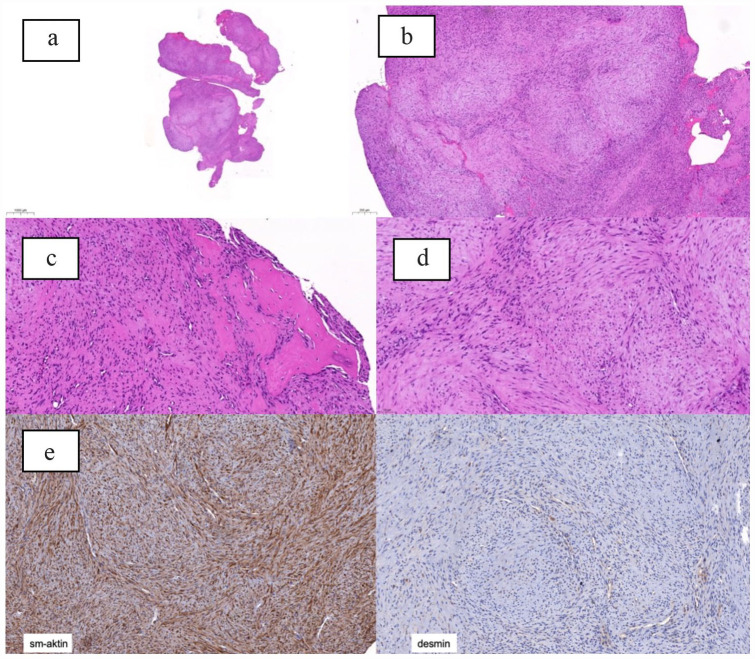
(a)–(d): spindle cell proliferation with multinodular growth pattern an alternating hypercellular and hypocellular areas. Mild nuclear atypia. Focal bone formation. (e): immune reactivity for sm-actin, negative for desmin.

After presenting the findings to our interdisciplinary tumor board, a biopsy was indicated. For this purpose, a CT-guided biopsy of the mass was performed. Histologically, there was a spindle cell lesion with mild nuclear atypia and pleomorphism with included trabecular bone. The immunohistochemical examination revealed a strong diffuse-positive immune reaction for vimentin, a nuclear-positive immune reaction for special AT-rich sequence-binding protein 2 (SATB2) (partial) and a positive immune reaction for smooth muscle actin (SMA) in the absence of the other markers (Pancytokeratin, S100, CD34, desmin, beta-catenin). Due to the only sparse sample material that was submitted, a further molecular pathological examination was not possible, so that a further classification of the lesion was not possible and an open re-biopsy was recommended. The samples obtained again showed an indicated biphasic neoplasia consisting of fascicular or cell-rich areas next to cell-poor, increasingly hyalinized, or chondroid-appearing areas without higher-grade cellular atypia, which was differentiated myofibroma-like from the histological aspect (Figure 2(a)–(d)). Mitoses were not clearly displayed. However, the performed Ki67 proliferation index showed an inhomogeneous proliferation activity with a Ki67 proliferation index of up to 15% detectable in the hotspots. The lesional cells only showed an immunohistological expression with the antibody against sm-actin. Furthermore, a (possibly unspecific) expression of SATB2 was also found. The remaining immunohistological tests (antibodies against beta-catenin, CD34, desmin, S100, STAT6) were negative again (Figure 2(e)). An insertion in exon 11 of PDGFRB (p.I538_L539insR) could be detected by molecular pathology. This variant was classified as probably activating in vitro (Figure 2(e)) so that in the absence of other tumors, the diagnosis of a solitary intraosseous myofibroma was finally made activating PDGFRB mutations represent a characteristic molecular alteration in these lesions. MDM2 amplification was not present, so that a low-grade osteosarcoma could be ruled out. There was no evidence of malignancy in the material available here.

### Therapy

Due to the lack of symptoms, the patient decided against surgical treatment. The CT follow-up after 3 months showed an unchanged lesion of constant size, which is why the patient continues to favor conservative therapy.

## Discussion

### Clinical findings

The authors present a case of an intraosseous myofibroma of the ribs resulting in pathological fracture. Intraosseous myofibromas are very rare, there is only a small number of case reports, which mostly describe a craniofascial localization. In 2009, Nirvikalpa and Narayanan^7^ identified fewer than 40 cases described in the literature since 1966. Since then, there have been mainly single case reports on patients with myofibromas of the mandible, temporal bone, orbit, and a literature review of six patients with myofibroma of the maxilla.^1,7–10^ In the international literature intraosseous myofibromas on the trunk or the extremities are very rare; there are often only single case reports on lesions on the clavicle (2 patients),^3,11^ spine (2 patients),^1^ ilium (1 patient),^12^ femur/hip (3 patients),^6,13^ tibia (3 patients),^2,4,14^ humerus (1 patient),^15^ and ulna (2 patients).^15,16^ Oudijk et al. analyzed 114 patients with myofbromas in 2012, only 8 lesions were intraosseous, 3 were located in the cranio-fascial area (mandibula, parietal bone), the others involving the clavicle, scapula, and tibia. All of these patients were younger than 25 years, 6 patients were even younger than 15 years.^3^ Yi et al.^6^ found 24 patients with myofibromas aged 8–64 years. The lesions were most frequently localized in the extremities (15 patients), followed by the head and trunk (6 patients each) and neck (2 patients), without further differentiation between intraosseous and subcutaneous locations.^6^ There is one previous report of a solitary myofibroma of the chest wall involving the rib.^17^

### Histological, immunohistochemical, and molecular findings

The list of possible differential diagnoses is long and mainly relates to benign and malignant spindle cell tumors. Histologically, myofibromas can easily be confused with neurofibromas, desmoid tumors, fibromatoses such as fibrous histiocytoma, solitary fibrous tumors/hemangiopericytomas, and various types of sarcomas.^1,2^

The literature research shows that intraosseous myofibromas are histologically often positive for SMA and muscle-specific actin (MSA) and other markers (S100, CD34) are often negative or only positive in a few cases (Desmin).^1,3^ The Ki67-index is often low, reflecting the low proliferation and slow growth progression of the lesions,^2^ which is consistent with our immunohistochemical results. With regard to the activating PDGFRB mutation, as it was found in our patient, the literature primarily contains associations with infantile myofibromatosis in children. Dachy et al.^18^ found gain-of-function mutations in samples from 25 children in their study with 69 patients, these were associated with severe multicentric diseases in 68%. Hung and Fletcher^19^ were able to demonstrate PDGFRB mutations not only in the context of myofibromatosis but also in conventional myofibromas in adult patients. Koo et al.^20^ found mutations in 8 out of 10 cases. Even though PDGFRB mutations may be detected more frequently in cases of multicentric lesions, the detection of this mutation may facilitate a difficult pathologic diagnosis and enable targeted therapies.^18,20^

### Therapy

With a benign lesion like this, there is basically no need for surgical/interventional therapy. However, it may be indicated if symptomatic complaints develop. In the literature, local surgical resection is usually described as the treatment of choice for complaints, which usually results in cure.^2,3^ Spontaneous remissions can occur in soft tissue and bone.^2^ The most common surgical procedure was complete excision, in two cases with replacement by an iliac bone grafting.^11^ In some cases, an intralesional curettage was performed without further defect reconstruction, occasionally a bone graft is described.^1,6,10^ A defect filling with cement or other adjuvants has not been reported. There were also a few patients without further therapy after biopsy.^6^

Recurrences have only been described in a few cases. In their case report, Ma et al.^1^ observed a recurrence of an intraosseous myofibroma on the finger after one year, Yi et al.^6^ reported 7 patients with recurrence in their case series of 24 patients and identified the absence of a pseudocapsule and younger age as possible risk factors for recurrence. Hung and Fletcher^19^ reported a patient with soft tissue myofibroma which recurred twice. Fu et al.^21^ described two recurrences of subcutaneous myofibromas. The surgical procedure has not been identified as a risk factor for recurrence in the current literature, possibly due to the small number of cases and the even lower number of reported recurrences. In our case, the patient refused therapy because her symptoms, which occurred temporarily due to the fall, had completely resolved. The CT follow-up after 1 year showed no size progression of the lesion, so that an operative therapy was not indicated until now.

## Conclusion

Intraosseous myofibromas are rare, benign neoplasms that can be easily confused with other benign and malignant spindle cell tumors clinically, radiologically, and histopathologically. Additional immunohistochemical and molecular pathological examinations such as SMA and a gain-of-function mutation in PDGFRB are reliable methods that enable a reliable diagnosis, so that unnecessarily aggressive therapy in benign tumors can be avoided. The prognosis for solitary lesions is excellent; for multicentric lesions, particularly in children with infantile myofibromatosis, the prognosis depends on the extent of the disease and the organs affected. Surgical therapy is indicated if patients complain of painful symptoms or pathological fracture. A complete excision of the lesion should then be performed, which usually results in lasting cure.
